# Improved dynamic monitoring of transcriptional activity during longitudinal analysis in the mouse brain

**DOI:** 10.1242/bio.037168

**Published:** 2018-10-19

**Authors:** Hwon Heo, Juyeong Jo, Jae In Jung, Young-Min Han, Seongsoo Lee, Song Rae Kim, Seung-Hae Kwon, Kil-Nam Kim, Byung Joon Hwang, Yun Kee, Byoung Dae Lee, Dongmin Kang, Song Her

**Affiliations:** 1 Western Seoul Center, Korea Basic Science Institute, Seoul 03759, South Korea; 2 Department of Life Science, Ewha Womans University, Seoul 03760, South Korea; 3 Chuncheon Center, Korea Basic Science Institute, Chuncheon 24341, South Korea; 4 Department of Molecular Bioscience, College of Biomedical Science, Kangwon National University, Chuncheon 24341, South Korea; 5 Division of Biomedical Convergence, College of Biomedical Science, Kangwon National University, Chuncheon 24341, South Korea; 6 Department of Physiology, School of Medicine, Kyung Hee University, Seoul, 02447, South Korea

**Keywords:** Pin-point monitoring transcription activity, Glucocorticoid receptor signaling, Luciferase, Brain promoter activity, Longitudinal monitoring, Stress

## Abstract

Bioluminescence imaging has proven to be a highly sensitive technique for assessing *in vitro* transcriptional activity toward understanding gene regulation patterns; however, application of this technique is limited for brain research. In particular, the poor spatiotemporal resolution is a major hurdle for monitoring the dynamic changes of transcriptional activity in specific regions of the brain during longitudinal analysis of living animals. To overcome this limitation, in this study, we modified a lentivirus-based luciferase glucocorticoid receptor (GR) reporter by inserting destabilizing sequence genes, and then the reporter was stereotaxically injected in the mouse infralimbic prefrontal cortex (IL-PFC). Using this strategy, we could successfully pin-point and monitor the dynamic changes in GR activity in IL-PFC during normal stress adaptation. The modified reporter showed a 1.5-fold increase in temporal resolution for monitoring GR activity compared to the control, with respect to the intra-individual coefficients of variation. This novel *in vivo* method has broad applications, as it is readily adaptable to different types of transcription factor arrays as well spanning wide target regions of the brain to other organs and tissues.

## Abstract (precis)

**Summary:** A platform for pin-point monitoring transcription activity in the deep brain of the mouse with high spatiotemporal resolution was developed, which is adaptable to various transcription factor arrays and targets.

## INTRODUCTION

Glucocorticoid receptors (GRs) are major players in the stress response, and impaired GR signaling is a postulated mechanism for the pathogenesis of stress-related mental disorders. One of the major challenges to overcome in research on stress is the individual variability, which can lead to grossly misleading conclusions owing to inconsistent results (Oitzl et al., 2010). In particular, this intra-individual variability can occur as a result of the conditions during set-point analysis for measuring stress responses, whereby an individual's stress responses may change cyclically over short periods. Nevertheless, most of the available information on *in vivo* GR signaling has been obtained from set-point studies, resulting in inconsistent results dependent on the time point of analysis. Therefore, it remains unclear whether these data from set-point studies faithfully reflect GR activity in the dynamic stress response. Thus, longitudinal analysis of GR signaling is critical to precisely understand neurobiological stress and its related mental disorders.

Bioluminescence imaging (BLI) is a powerful technique, which has been applied in a wide variety of biological fields, including reproduction (Kim et al., 2010), tumor growth and metastasis (Lee et al., 2012a), drug delivery (Nam et al., 2009), drug pharmacokinetics and biodistribution (Lee et al., 2011), and gene therapy, with unique advantages over conventional fluorescent reporters. The application of BLI has even extended to the *in vivo* study of transcriptional activity using a bioluminescent reporter. Although bioluminescence from the mouse brain has been imaged non-invasively through the intact skull, monitoring of transcriptional activity in the brain has largely been restricted to only precisely evaluating the activity due to the poor spatiotemporal resolution of BLI in this region. Indeed, many researchers have developed transgenic mouse models for studying *in vivo* transcriptional activity, but the clonal ubiquity leads to the poor spatial resolution that severely impedes the pin-point monitoring of transcriptional activity in targeted organs or tissues. To solve this problem of a ubiquitous bioluminescence signal, a Cre-Lox recombination system developed to generate a transgenic mouse that can effectively monitor the transcriptional activity of specific cell types (Akhmedov et al., 2016). Waddington's group also introduced a novel approach for developing a tissue-targeted delivery system to neonatal rodents, which served as an improved cell type-specific assay (Buckley et al., 2015). However, these methods still suffer from unsolved problems of the broadly distributed bioluminescence across targeted cells or organs. In particular, this poor spatial resolution of bioluminescence is a major disadvantage to study anatomical brain function of different regions of the brain, or even for the same cell type. Moreover, these transgenic strains are often developed and maintained on a limited number of genetic backgrounds, and therefore backcrossing is frequently necessary, leading to increased costs and significant time delays for research output.

**Fig. 1.**
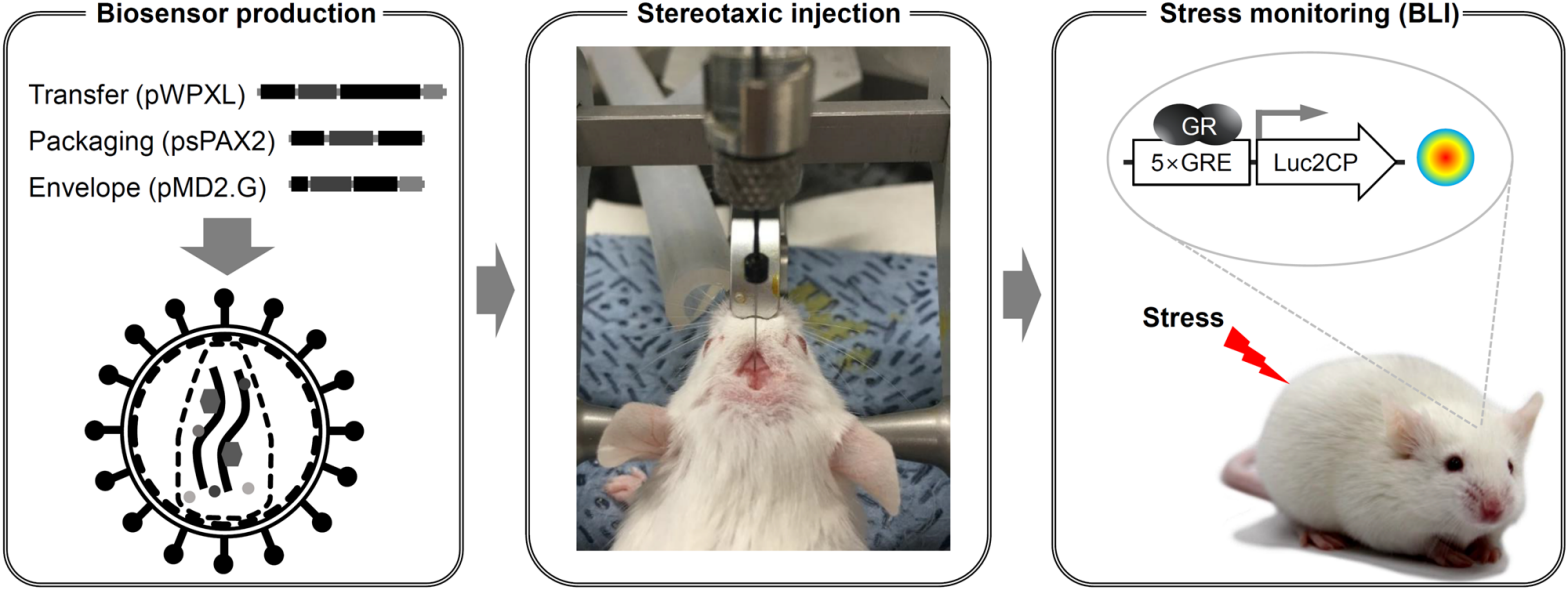
**Schematic illustration of stress monitoring in the brain of a living mouse.** A lentivirus-based luciferase GR reporter was stereotaxically micro-injected into the infralimbic area of the prefrontal cortex of the brains of CD-1 mice. BLI was performed to monitor GR activity following 2 h of restrained stress using an IVIS Spectrum system. BLI, bioluminescence imaging; GR, glucocorticoid receptor; 5×GRE, 5× glucocorticoid response elements; Luc2CP, luciferase2 hCL1/hPEST.

We previously reported a method for the successful pin-point monitoring of dynamic changes in GR activity in the rat hippocampal CA1 region using stereotaxic microinjection with a lentivirus-based luciferase GR reporter (Lee et al., 2016). The biosensor is composed of an insulator, and the firefly luciferase gene (Luc) under the control of five repetitive glucocorticoid responsive elements (5×GRE) within a minimal promoter (Lee et al., 2016). Using this approach, we found that the temporal variability of hippocampal GR activity was significantly correlated with depressive behaviors. Despite the improved spatial resolution, the reporter's temporal resolution was still somewhat limited. Low temporal resolution due to the prolonged half-life of luciferase increases the time intervals required for monitoring GR activation, which leads to ambiguous readouts of temporal variability. Therefore, the success of longitudinal monitoring of transcriptional activity requires a luciferase reporter with a short half-life. In this study, we introduced destabilization motifs (hCL and PEST; Leclerc et al., 2000; Li et al., 1998) for luciferase used in the stereotaxic micro-injection strategy to improve the spatio-temporal resolution of BLI analysis (Fig. 1). We evaluated the feasibility of our strategy by monitoring dynamic changes in GR activity in the infralimbic prefrontal cortex (IL-PFC) of the mouse brain during stress adaptation.

## RESULTS

### Luc2 enhanced GR activity

**Fig. 2.**
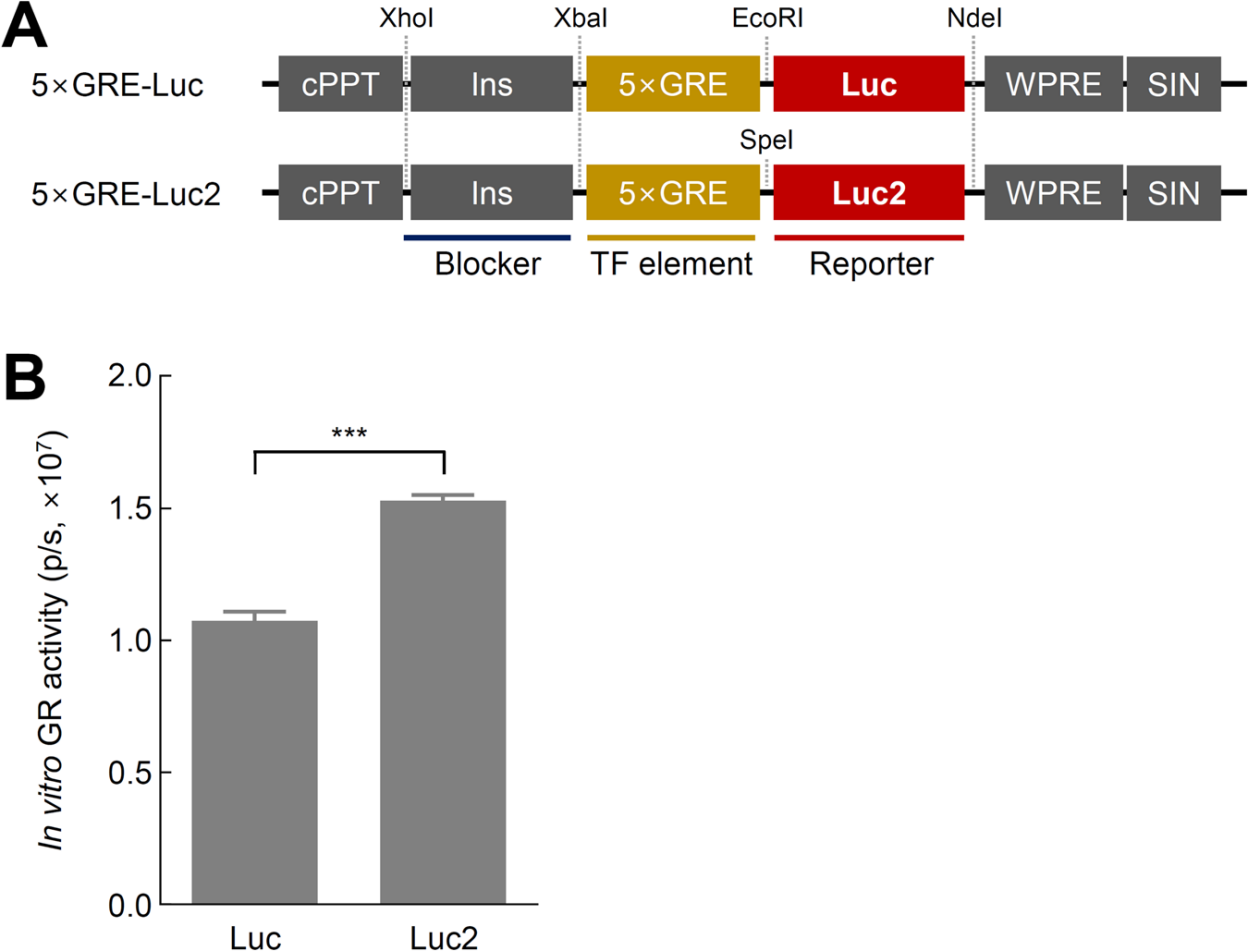
**Effects of the Luc2 sequence on the signal intensity in cultured H19-7 cells.** (A) Schematic diagram of the Luc and Luc2 reporter genes. (B) GR activity of reporters induced by treatment with 500 nM CORT (*n*=5; two sample *t*-test, ***P<0.001 versus Luc). The data represent the mean±s.e.m. 5×GRE, 5× glucocorticoid response element; Luc, luciferase; CORT; corticosterone; p/s, p/s/cm^2^/sr.

We initially modified our previously reported lentivirus-based luciferase GR reporter (5xGRE Luc reporter) by replacing Luc with Luc2 in the transfer vectors (Fig. 2A) to increase the signal intensity of luciferase. Luc2 results in a shorter half-life of luciferase to achieve high spatial resolution owing to the decreased amounts of luciferase, resulting in lower BLI signals (Luker and Luker, 2008). Stress was induced by corticosterone (CORT) treatment, and the induced GR activity was measured in H19-7 immortalized rat hippocampal neural progenitor cells. Indeed, a 1.4-fold greater GR activity level was found using the Luc2 reporter than with the conventional Luc reporter (Fig. 2B; T_4_=11.14, *P*<0.001). These data are consistent with previous findings demonstrating that Luc2 is the brightest luciferase available (Mezzanotte et al., 2013).

### Destabilizing the reporter sequences shortened the half-life of GR activity

**Fig. 3.**
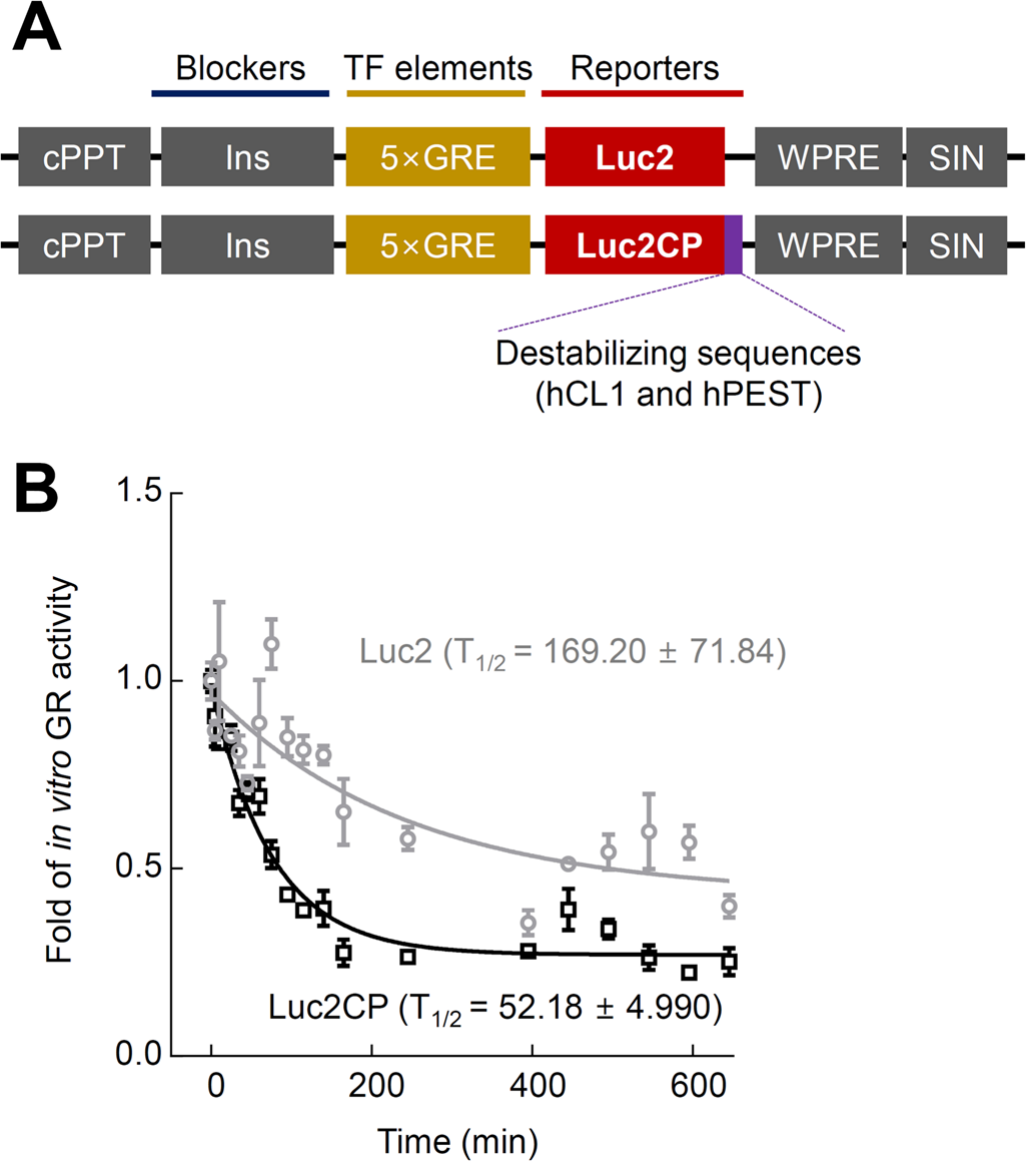
**Effects of the addition of destabilizing CP sequences in Luc2 on the half-life (T_1/2_) of luciferase activity in cultured H19-7 cells.** (A) Schematic diagram of the stable and destabilized reporter genes. The Luc2CP reporter contains the destabilizing CP sequences. (B) Luciferase activity of reporters induced by 500 nM CORT treatment (*n*=5) and subsequently incubated with 100 μg/ml cycloheximide to block the synthesis of new proteins. The data represent the mean±s.e.m. The fitted curve and half-life of luciferase activity were obtained using nonlinear regression analyses (one-phase decay). 5×GRE, 5× glucocorticoid response elements; Luc2, luciferase 2; Luc2CP, luciferase 2 hCL1/hPEST; CORT, corticosterone.

To construct a reporter with a faster turnover, we inserted the destabilizing CP sequences (hCL1 and hPEST) to the C-terminus of Luc2 (Fig. 3A). *In vitro* half-life analyses of GR activity with cycloheximide treatment showed a time-dependent decrease in GR activity of both the destabilized (Luc2CP) and original Luc2 reporter. However, the half-life of the Luc2CP reporter (52.18±4.99 min) was threefold shorter than that of the Luc2 reporter (169.20±71.84 min, Fig. 3B).

### Longitudinal monitoring of dynamic GR activation in response to acute stress

**Fig. 4.**
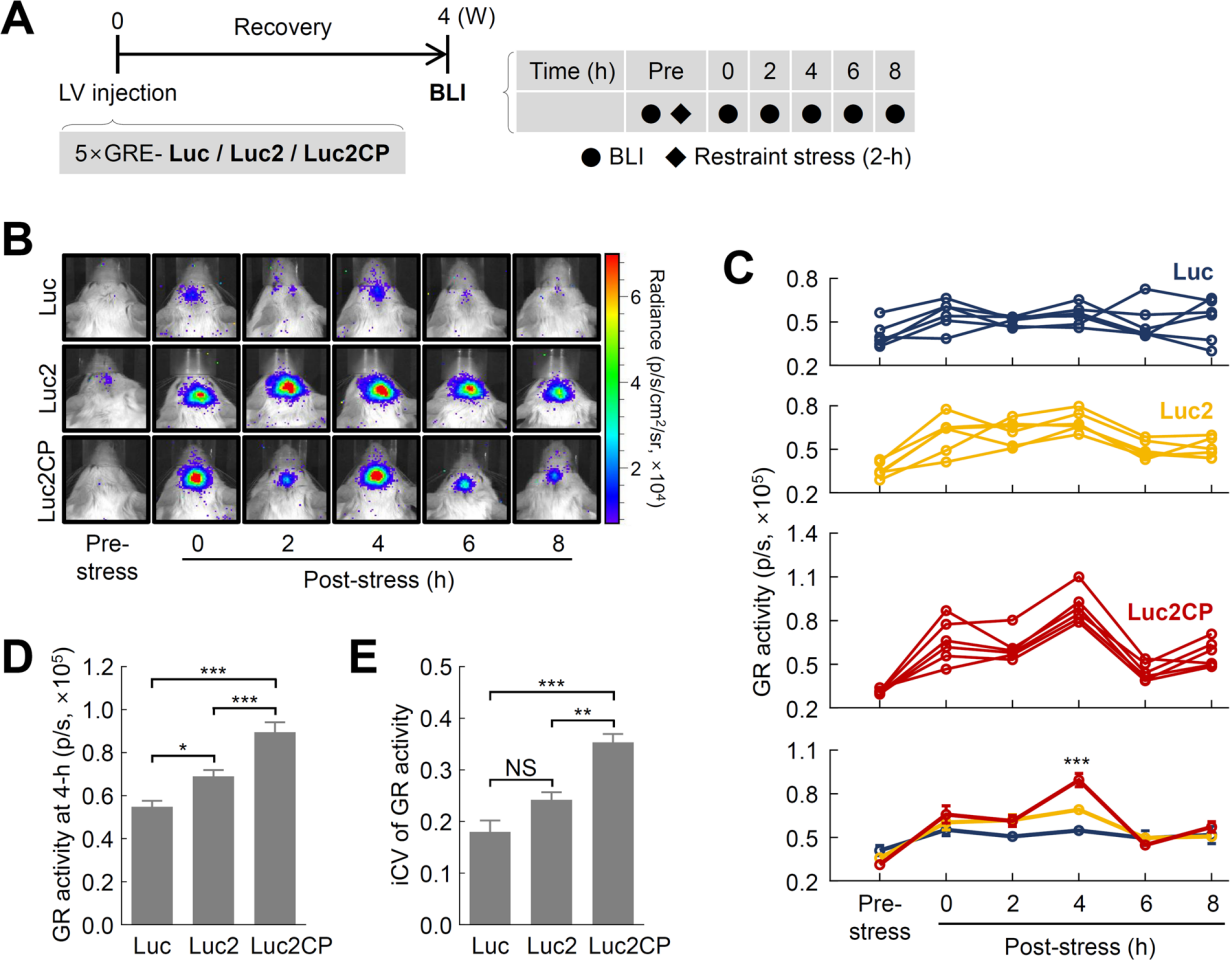
**Effects of three 5×GRE reporters on the temporal dynamics of GR activity in the IL-PFC of living mice.** (A) Schematic diagram of the experimental design. (B) Representative *in vivo* BLIs of the mice stereotaxically injected with the reporters (Luc, Luc2, and Luc2CP) in the IL-PFC, respectively. (C) Individual profiles (*n*=6) of BLI signals by Luc, Luc2, and Luc2CP, and the averaged signal of GR activity. ***P<0.001 versus Luc. (D) Comparison of GR activity at 4 h post-stress. (E) iCV over the six time points, including pre-stress imaging (one-way ANOVA with Bonferroni’s post-hoc test). The data represent the mean±s.e.m. *P<0.05, **P<0.01, ***P<0.001. 5×GRE, 5× glucocorticoid response elements; Luc2, Luciferase2; Luc2CP, Luciferase2 hCL1/hPEST; iCV, intra individual coefficients of variation; BLI, bioluminescence imaging; p/s, p/s/cm^2^/sr.

Next, we evaluated whether the modified reporter can be used to monitor dynamic changes in brain GR activity during stress adaptation. The mice were stereotaxically injected with the three 5×GRE reporters (Luc, Luc2, and Luc2CP) into the right IL-PFC, and then stress-induced GR activation was monitored over six time points, beginning prior to the stress exposure (Fig. 4A). As shown in Fig. 4B, a pulsatile pattern of GR activity was found in the mice injected with the modified 5×GRE-Luc2CP reporter after 2 h of stress, whereas there was no pulsatile pattern detected in the groups injected with the other two 5×GRE reporters (Luc and Luc2). Furthermore, the conventional reporter (Luc) showed much weaker GR activities compared with those of the two other reporters. Two-way analysis of variance (ANOVA) indicated that GR activity significantly changed over time and differed according to reporter type (Fig. 4C; time: *F*_5,90_=29.270, *P*<0.001; group: *F*_2,90_=6.368, *P*<0.01; group×time: *F*_10,90_=4.488, *P*<0.001). *Post-hoc* analyses further confirmed significant differences in time-dependent GR activity between Luc2 and Luc2CP at 4 h post-stress (Fig. 4D; T_90_=3.795, *P*<0.001). Comparison of the intra-individual coefficient of variation (iCV) also showed that the temporal variability of the Luc2CP reporter was 1.5-fold greater than that of the Luc2 reporter (Fig. 4E; one-way ANOVA, *F*_2,15_=23.310, *P*<0.001; T_15_=4.328, *P*<0.005), with no significant differences in iCV between the Luc and Luc2 reporters (Fig. 4E; T_15_=2.410, *P*=0.088). These results suggest that insertion of the destabilizing sequences and stereotaxic microinjection strategy provides high temporal resolution allowing us to monitor more dynamic changes in GR activity during stress adaptation.

### Regional differences in GR expression

**Fig. 5.**
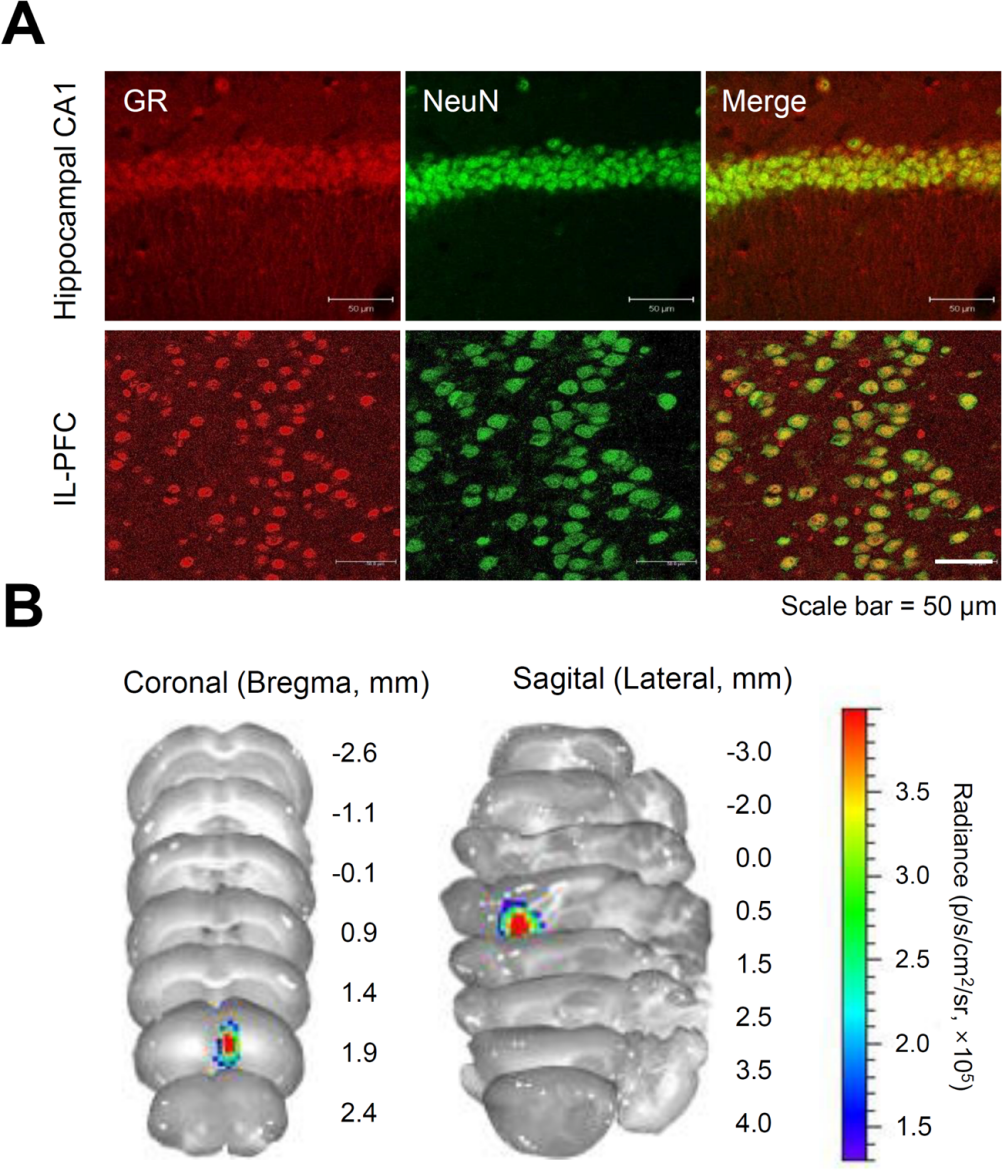
**Regional differences in GR expression.** (A) Representative confocal imaging of the GR anatomical distribution in the hippocampal CA1 region and IL-PFC. Mice that did not receive any treatment were euthanized for immunohistochemical assessment. Tissue sections were stained with GR (red) and NeuN (green) antibodies to determine the location of GR expression. (B) Representative ex vivo BLIs in serial sections obtained 25 min following stress and intraperitoneal luciferin injection. BLI signals were detected at the site of the stereotaxic injection.

Immunohistochemistry and confocal imaging confirmed a differential GR expression in which the dense distribution pattern for GR expression in the mouse hippocampal CA1, whereas low immune-reactivity was found in the IL-PFC (Fig. 5A). Dual-labeling immunohistochemistry with NeuN showed that GRs were expressed in the NeuN+ neuronal cells of both regions. The anatomical accuracy of GR activation was confirmed by *ex vivo* BLI in IL-PFC (Fig. 5B).

On the basis of these results, it was concluded that GR activity is likely differential between the two regions although occurring in the same NeuN+ neuronal cells, thereby highlighting the importance of the pin-point monitoring of GR activity.

## DISCUSSION

Using a combination of a modified lentiviral luciferase reporter and stereotaxic microinjection, we successfully demonstrated robust and dynamic brain GR activation in response to acute stress in mice. The main strength of this study is the achievement of dynamic monitoring of brain GR activity with a luciferase reporter tagged with destabilization motifs. Our strategy shows several other advantages over conventional methods. Importantly, it allows for anatomical pin-point analysis by stereotaxic microinjection of the reporter, which is applicable to animals other than mice. Moreover, the lentivirus-based transgene production of animal models is a much cheaper and more rapid process compared to the establishment of transgenic mice. The lentiviral-based transgene also shows long-term expression compared to an adenoviral-based transgene (Nayak and Herzog, 2010), and induces less of an inflammatory response than an adenovirus (Felizardo et al., 2011). Nevertheless, since the recombinant lentiviral vectors do still induce minimal inflammatory responses by removing over 95% of the parental viral genome, it is necessary to exert experimental caution by performing analysis of inflammation reactions.

We previously developed a lentivirus-based luciferase biosensor for the longitudinal analysis of GR activity. We monitored dynamic GR activation in the rat hippocampal CA1 after each 2-h exposure to acute stress after stereotaxic microinjection of the reporter. Although the reporter showed clear GR activation in the rat hippocampal CA1, the present results in murine IL-PFC showed only a weak signal of GR activation. Two possibilities for these observations can be proposed. The first is related to the different depth of bioluminescence signals since the dorsal hippocampal CA1 (anterior −1.88 mm, lateral −1.60 mm, ventral −1.30 mm) is deeper than the murine IL-PFC (anterior +1.95 mm, lateral −0.40 mm, ventral −3.10 mm). Indeed, bioluminescence is dependent on the physical constraints of light scattering and absorption in deep brain tissues (Aswendt et al., 2017). The second possibility is the differential expression pattern of GR. It is well-known that GR shows distinct expression patterns in different brain regions, although it is constitutively expressed (Vandevyver et al., 2014). As shown in Fig. 5, the GR expression pattern of hippocampal CA1 is denser than that of IL-PFC, which may influence the signal intensity derived from GR-expressing targeted cells.

Similar to previous findings (Leclerc et al., 2000; Li et al., 1998), a short half-life of luciferase was achieved by inserting the destabilizing sequence CP to the C-terminal of luciferase (*in vitro* T_1/2_=52.18±4.99 min). This short half-life resulted in high temporal resolution that was sufficient to discern a pattern of GR activity at 2-h time intervals of BLI during stress adaptation. In general, normal firefly luciferase has a half-life of 3–4 h, which can be suitable for studies requiring only once-daily assays over the course of several days, such as assessment of viral infection and treatment effects (Luker and Luker, 2009). This short half-life yielded poor signal intensities of luciferase, which has made it very difficult to study particularly deep brain regions. The further modified reporter described herein eliminates this disadvantage by replacing the Luc gene with Luc2, which is codon-optimized for expression in mammalian cells (Mašek et al., 2013). As expected, Luc2 yielded brighter bioluminescence signals induced by acute stress at 4 h post-stress. Unexpectedly, however, the destabilizing CP sequence significantly increased the bioluminescence signals as demonstrated by a 1.3-fold higher signal intensity of the Luc2CP reporter than that of the Luc2 reporter at 4 h post-stress. This suggests that the enhanced temporal resolution conferred by the destabilizing sequence CP allowed for episodic synchronization of GR activity, revealing an evident peak at 4 h post-stress. These results highlight the importance of achieving high temporal resolution for the dynamic monitoring of neurobiological processes in the brain.

Interestingly, our GRE-Luc reporter yielded weaker bioluminescence signals (∼1×10^5^ p/s/cm^2^/sr in 1-min exposures), with signal intensity more than two orders of magnitude lower, in the brains compared with that reported for a *Cre*-transgenic mouse imaged using similar instrument settings (∼2×10^7^ p/s/cm^2^/sr in 5-sec exposures; Akhmedov et al., 2016). This difference in signal intensity may be explained by different experimental conditions such as the different exposure time of BLI and the use of different transcription factor-binding arrays. However, the weak signal intensity may be related to the different animal model used in our study. We stereotactically injected a small amount of the reporter (1 μl) into the targeted brain region. Despite the relatively weaker signals, the intensity of BLI was nevertheless sufficient to monitor bioluminescence signals generated from the mouse IL-PFC during stress adaptation. Furthermore, the stereotactic procedure provides the added advantage of investigating neuroanatomical links between specific small areas of the prefrontal cortex such as a neurobiological interaction between the IL-PFC and prelimbic prefrontal cortex (PrL-PFC) in which GRs are expressed (Drevets et al., 2008; Myers-Schulz and Koenigs, 2012; van Aerde et al., 2008). The PrL-PFC is linked to the nucleus accumbens and basolateral amygdala, and plays a major role in control of stress response inhibition and reward. The IL-PFC is connected to visceral/emotional effector systems (i.e. the central amygdaloid nucleus and hindbrain cardiovascular regulatory pathways) and is important for the control of emotional responses to fear as well as activation of stress effector pathways (Vertes, 2004).

In conclusion, to our knowledge, this is the first report demonstrating the monitoring of GR signals during the entire stress adaptation process in the mouse IL-PFC. Employing our methodology as a platform to study GR activity might open new possibilities to elucidate the spatio-temporal dimensions of the molecular changes occurring upon GR activation during the process of stress-related mental disorders, such as post-traumatic stress disorder and depression. We also anticipate using this system to address additional pharmacological and physiological challenges, and to test the utility of this platform for monitoring other dynamic transcription factor activities in organs other than the brain.

## MATERIALS AND METHODS

### Preparation of lentiviral constructs and production of the lentivirus

Luciferase genes (Luc) from pGL4.10[luc2] or pGL4.12[luc2CP] (Promega, Madison, USA) were replaced with the Luc sensor region of the pWPXL: 5×GRE-Luc lentiviral transfer vector (Lee et al., 2016). The elongation factor 1α (EF1α) promoter was replaced by the 5×GRE sequences of the 5×GRE-Luc2CP lentiviral transfer vectors to serve as a control reporter constitutively expressing luciferase. The lentivirus was produced by co-transfecting the transfer vector, second-generation packaging vector (psPAX2, Addgene plasmid 12260), and an envelope plasmid (pMD2.G, Addgene plasmid 12259) at a ratio of 2:1:1 into HEK-293T cells [American Type Culture Collection (ATCC), Manassas, USA] using the Effectene reagent (Qiagen). Viral supernatants were collected 48 h after transfection, passed through a 0.2-μm filter, and concentrated by centrifugation using PEG-itTM virus precipitation solution (System Biosciences, Palo Alto, USA). Aliquots were stored in phosphate-buffered saline (PBS) in the presence of TransDux^TM^ MAX Lentivirus Transduction Enhancer (System Biosciences) and stored at −80°C until use. The titers of lentivirus were in the range of 1.50–1.95×10^12^ genome copies/ml.

### H19-7 cell culture and half-life assay of luciferase reporters

Immortalized hippocampal neurons (H19-7) from rats were purchased from ATCC. The cells were maintained in Dulbecco's modified Eagle's medium (Thermo Fisher Scientific) containing 10% fetal bovine serum (Thermo Fisher Scientific) and 100 U/ml penicillin/streptomycin (Thermo Fisher Scientific) at 34°C in a 5% CO_2_ incubator. For the luciferase activity test, the H19-7 cells were seeded into 96-well plates at a density of 1×10 cells per well (Fig. 1B). The next day, the cells were infected with the 5×GRE-Luc or 5×GRE-Luc2 vector, and incubated for 36 h in the presence of 500 nM CORT (Sigma-Aldrich). After the CORT treatment, D-luciferin (final concentration=150 μg/ml, Gold Biotechnology, St. Louis, USA) was added to each well and the luciferase activity was measured using an IVIS® Spectrum *in vivo* imaging system (PerkinElmer Inc., Waltham, USA). For the half-life assay of the luciferase reporter (Fig. 2B), H19-7 cells were cultured in 96-well plates at a density of 1×10^4^ cells per well. The following day, the cells were infected with 5×GRE-Luc2 or 5×GRE-Luc2CP lentiviral vectors. 12 h after infection, the cells were treated with CORT to induce reporter expression, and 36 h later, cycloheximide (100 μg/ml, Sigma-Aldrich) was added to the media at designated intervals over 700 min (for a generating modified logarithmic scale: 0, 5, 10, 25, 35, 45, 60, 75, 95, 115, 140, 165, 245, 395, 445, 495, 545, 595 and 645 min). D-luciferin was then added to each well again, and the luciferase activity was measured using the IVIS system.

### Immunofluorescence and confocal imaging

The brain was sectioned coronally (30-μm thick) and processed for immunofluorescence staining with primary antibodies specific for GR (1:500; Santa Cruz Biotechnology) and neuronal nuclei (NeuN; 1:500; Santa Cruz Biotechnology). Images were acquired on a confocal laser-scanning microscope (LSM-780 NLO; Carl Zeiss, Oberkochen, Germany) equipped with a C-Apochromat 20×/0.5 W objective lens.

### Animal care

Male CD-1 mice (25–30 g, 5 weeks old) were purchased from Daehan Biolink Co. Ltd. (Taconic Biosciences, Seoul, Korea), and housed at the Korea Basic Science Institute (KBSI) Animal Care Unit for 1 week prior to experiments. The animals were individually housed in transparent plastic cages with wire grid covers under controlled temperatures (22–24°C) with a 12-h light:dark cycle (photoperiod from 08:00 to 20:00). The KBSI Animal Care and Use Committee approved this study (KBSI-AEC 1404), and all animal procedures were conducted in accordance with the KBSI Guide for the Care and Use of Laboratory Animals.

### Stereotaxic microinjection of luciferase reporters

The mice were anesthetized in an induction chamber with 2.5% isoflurane in 100% oxygen at a flow rate of 1.0 liter/min for 5 min and placed into a stereotaxic apparatus (Kopf Instruments, Tujunga, USA) with gas anesthesia masks (1.0% isoflurane in 100% oxygen at 0.5 liter/min). The lentiviral luciferase reporters (EF1α-Luc2, EF1α-Luc2CP, 5×GRE-Luc, 5×GRE-Luc2, and 5×GRE-Luc2CP titer 1.69×10^12^) were injected into the right IL-PFC based on coordinates from the bregma and dura mater according to Allen Mouse Brain Atlas (Lein et al., 2007) (anterior +1.95 mm, lateral −0.40 mm, ventral −3.10 mm). A 1-µl viral solution was injected at a rate of 0.25 µl/min using a micro-syringe nanopump (Leica Microsystems, Concord, Canada) and a 5-µl Hamilton syringe. The syringe was held in place for 10 min before withdrawal. The mice were allowed at least 4 weeks of recovery in their home cages before the imaging experiments.

### Imaging of *in vivo* luciferase activity

The *in vivo* visualization of luciferase activity using the BLI technique was conducted according to our previous reports (Lee and Her, 2013; Lee et al., 2012a; Lee et al., 2011; Lee et al., 2010; Lee et al., 2012b). In brief, all mice received an intraperitoneal injection of 100 mg/kg D-luciferin dissolved in Dulbecco's PBS and then were anesthetized in an induction chamber with 2.5% isoflurane in 100% oxygen at a flow rate of 1.0 liter/min for 10 min. For *in vivo* BLI analyses, the mice were imaged simultaneously for 1-min exposures using the IVIS with a 2.0% mixture at 0.5 liter/min, and the regions of interest were quantified with photon flux (p/s) using the IVIS. The data represent BLI signals from an individual mouse combined from two independent experiments. In accumulation tests, BLIs were monitored for 2 h. For the accumulation of bioluminescent signals, D-luciferin was injected twice at 15 min before acquisition of BLIs (0 and 2 h). For the monitoring of *in vivo* GR activity, mice injected with the 5×GRE-containing lentiviral reporter were subjected to restraint stress for 2 h. BLI was performed immediately thereafter (0 h) and monitored at 2-h intervals for a total of 10 h (pre-stress, 0, 2, 4, 6, and 8 h post-stress). For the *ex vivo* BLI analyses, after completing the acute stress, mice were injected with luciferin 15 min before euthanization. The brain was removed and dissected into about 1-mm coronal or sagittal segments using a slicer matrix (ASI Instruments, Warren, USA).

### Statistical analyses

The data are expressed as mean±s.e.m. Statistical analyses were performed using Prism 4 software (GraphPad Software, La Jolla, USA). One-way ANOVA, followed by Bonferroni's *post-hoc* analyses, were used to compare the luciferase activity in cultured H19-7 cells, the GR activity at 4 h after stress, and the iCV value of GR activity, Two-way repeated-measures ANOVA was used for analysis of the data from other experiments. Temporal variation in BLI signals was determined according to the iCV values as previously described (Lee et al., 2016). The iCV reflects the extent of variation in the individual data sets during imaging periods, calculated as the standard deviation of individual BLIs divided by the mean value over the 10-h imaging duration.
